# Buckwheat and CVD Risk Markers: A Systematic Review and Meta-Analysis

**DOI:** 10.3390/nu10050619

**Published:** 2018-05-15

**Authors:** Liangkui Li, Georg Lietz, Chris Seal

**Affiliations:** Human Nutrition Research Centre, Institute of Cellular Medicine, Faculty of Medical Sciences, Newcastle University, Newcastle upon Tyne NE2 4HH, UK; Liangkui826@outlook.com (L.L.); Georg.Lietz@ncl.ac.uk (G.L.)

**Keywords:** buckwheat, CVD risk markers, meta-analysis

## Abstract

The effects of buckwheat intake on cardiovascular diseases (CVDs) have not been systematically investigated. The aim of the present study was to comprehensively summarize studies in humans and animals, evaluating the impact of buckwheat consumption on CVD risk markers and to conduct a meta-analysis of relevant data. Thirteen randomized, controlled human studies, two cross-sectional human studies and twenty-one animal studies were identified. Using random-effects models, the weighted mean difference of post-intervention concentrations of blood glucose, total cholesterol and triglycerides were significantly decreased following buckwheat intervention compared with controls [differences in blood glucose: −0.85 mmol/L (95% CI: −1.31, −0.39), total cholesterol: 0.50 mmol/L (95% CI: −0.80, −0.20) and triglycerides: 0.25 mmol/L (95% CI: −0.49, −0.02)]. Responses of a similar magnitude were seen in two cross-sectional studies. For animal studies, nineteen of twenty-one studies showed a significant reduction in total cholesterol of between 12% and 54%, and fourteen of twenty studies showed a significant reduction in triglycerides of between 2% and 74%. All exhibited high unexplained heterogeneity. There was inconsistency in HDL cholesterol outcomes in both human and animal studies. It remains unclear whether increased buckwheat intake significantly benefits other markers of CVD risk, such as weight, blood pressure, insulin, and LDL-cholesterol, and underlying mechanisms responsible for any effects are unclear.

## 1. Introduction

Across the globe, cardiovascular diseases (CVDs) are the leading cause of morbidity and death, and account for approximately one-third of all deaths around the world [1]. Elevated blood pressure, raised total cholesterol, low density lipoprotein cholesterol (LDL-cholesterol) and high density lipoprotein cholesterol (HDL-cholesterol) concentrations are clinically considered as major CVD risk factors. There are increasing epidemiological studies suggesting that diets rich in whole grains are linked to a lower risk of CVD and mortality [2,3,4,5,6]. In China, recent changes to traditional diets, which have shown a dramatic decrease in the amount of whole grain consumption from 104 g/day in 1982 to 24 g/day in 2002, may be a contributory factor for the elevated CVD mortality in this country [6,7]. The pseudo-cereal buckwheat, which belongs to the Polygonaceae family, is included in the “whole grain” category in terms of nutritional value [8]. Buckwheat has been cultivated as a traditional food in China since 1000BC and is found almost everywhere globally, but mainly in the northern hemisphere, such as in Russia and China [9].

In recent years, there has been increasing interest in the use of buckwheat as a raw food material owing to its “re-discovered” nutritional value and health benefits [9,10]. Among the main nine species with agricultural significance, common buckwheat and Tartary buckwheat (also known as bitter buckwheat) are the most widely grown species [11]. Buckwheat seeds are the principle form for human consumption, and they are mainly consumed as milled flours used in bakery products (bread, noodles, snacks and cookies) enriched with buckwheat flour at levels ranging up to 60%, and in non-bakery buckwheat products (honey, tea and sprouted grains) [12]. In addition to a high starch content as an energy source, buckwheat is rich in nutritionally valuable protein with a well-balanced amino acid profile, dietary fibre, lipids and minerals, along with other health-promoting components such as phenolic compounds and sterols, which have attracted growing attention to buckwheat as a potential functional food [13]. Buckwheat, as a traditional Chinese foodstuff, is well known to contain high concentrations of rutin compared with other common plant foods. In addition, the absence of gluten makes buckwheat-containing products potential alternatives for patients suffering from celiac disease [14]. It has been demonstrated that intake of buckwheat or buckwheat-enriched products is associated with a wide range of health benefits, including anticancer, anti-inflammatory, hypoglycemic and hypocholesterolemic effects, although the specific bioactive components responsible for the beneficial effects of buckwheat remain uncertain [15].

To date, relatively few studies have been carried out to investigate the impact of buckwheat intake on human health. Moreover, to our knowledge, there has not been any quantitative study to systematically review and summarize the effects of buckwheat consumption on CVD risk markers. With accumulating evidence, the object of this work was to comprehensively review the recent literature and carry out a meta-analysis evaluating the changes in blood glucose and lipid concentrations induced by buckwheat intake in humans and animals. A secondary objective was to explore possible mechanisms underlying any beneficial effects observed.

## 2. Materials and Methods

### 2.1. Data Sources and Literature Search

A comprehensive literature search for prospective studies that had evaluated the correlation between buckwheat intake and CVD risk between 1960 and 2018 was undertaken. PubMed, Scopus, Ovid, EBSCO, Web of Science, ProQuest databases, Science, JSTOR, Medline and China National Knowledge Infrastructure were searched using the search terms ‘buckwheat’ AND ‘cardiovascular disease’ OR ‘cholesterol’ AND ‘human’ OR ‘animal’, and the same terms were applied in each database during the search phase. CVD was defined to include stroke, aortic disease, peripheral arterial disease and coronary heart disease. In addition, the reference lists of retrieved papers were searched manually for all additional potentially relevant papers. The search was restricted to studies on humans and animals and included those that were written in different languages including English or Chinese. Data were extracted by a single reviewer.

The studies included in this review met the following criteria: (1) a prospective cohort study, (2) normal laboratory animals or free living humans, (3) buckwheat-intake exposure, (4) the results included markers of CVD risk, such as plasma glucose and insulin concentrations and lipid profile. Since cholesterol was the most common indicator of CVD response to whole-grain foods, cholesterol was used as a primary outcome marker in this review. The eligibility criteria were set before the start of the research.

### 2.2. Data Extraction

The following data were extracted from each human study: lead author, year of publication, characteristics of subjects, number of subjects, mean/median intake of buckwheat, types of buckwheat consumed, trial length and findings. The sample size of human studies in this review was the overall total for the experiment rather than restricting to either control or intervention diet/s. The following data were extracted from each animal study: lead author, year of publication, animal species, mean/median intake of buckwheat, experimental diet, trial length and outcomes. Missing data are reported as ‘‘Not stated’’ if they were not explained in the corresponding articles.

### 2.3. Statistical Analysis

All statistical analyses were performed with STATA 12.0 (Stata Corp, College Station, TX, USA); *P* < 0.05 was considered significant. Heterogeneity across studies was quantified by using the *I*^2^ statistic to consider each study design, as a quantitative evaluation of inconsistency among studies [16]. To pool the results of studies of the acute impacts on blood glucose, lipid profiles, a fixed effects model was used when heterogeneity was absent or low (*I*^2^ < 20%); when heterogeneity was greater, a random-effects model was used. In this review, weighted mean differences (WMDs) between treatment (buckwheat diet) and control groups (normal or refined diet) or before and after treatment were combined via a random-effects model to evaluate the size of treatment impacts on CVD risk markers, including blood concentrations of glucose, total, HDL and LDL cholesterol and triglycerides. To examine whether a single study exerted undue impact on the overall results, sensitivity analyses were performed in which each individual study was excluded from the meta-analysis and the effect size recalculated with the remaining studies. For all outcomes, a priori subgroup analyses were planned to be conducted with meta-regression models, if there were ≥10 studies. Results of the studies reported in mg/dL were converted to mmol/L using standard conversion factors, with 1 mg/dL = 0.02586 mmol/L for cholesterol, 1 mg/dL = 0.01129 mmol/L for triglycerides. These values were obtained as mean ± SD. For continuous results, summary estimates of WMD with 95% CI were assessed for net changes between each treatment and control groups. Finally, potential publication bias of the studies was also evaluated by visual inspection of Funnel plots and quantitatively assessed using Begg’s and Egger’s tests, where *P* < 0.05 was deemed statistically significant [17].

## 3. Results

### 3.1. Study Selection

**Figure 1:**
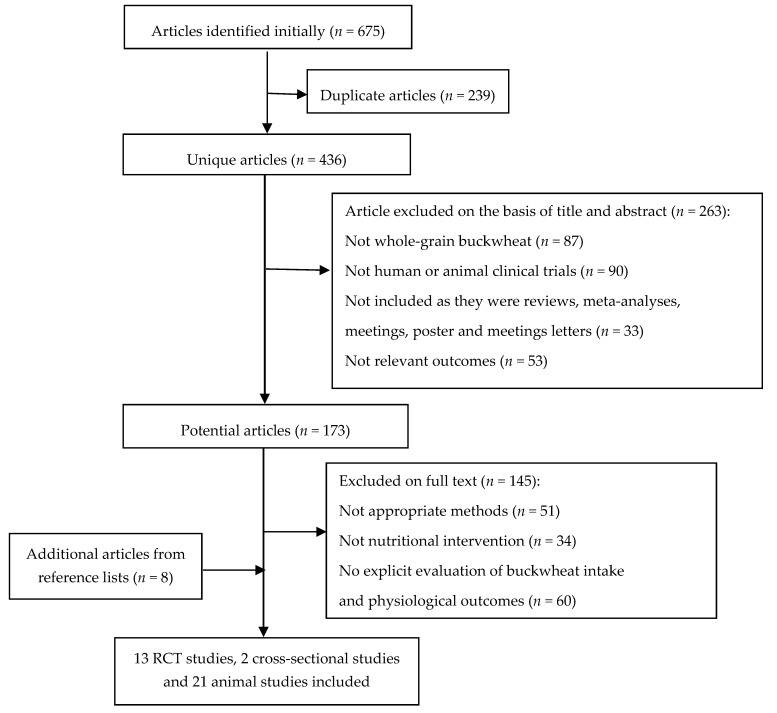
Flow diagram of article selection.

As shown in Figure 1, the systematic search of the scientific databases led to the initial identification of 675 articles for further evaluation. After removing duplicate articles (239) and articles that did not meet the eligibility criteria (408), a total of 28 articles including 11 human studies and 17 animal studies were included in the review. It was noteworthy that five trials were reported in the same population; thus, the current review combined the informative data and retained only the latest paper to avoid information duplication [18,19,20,21,22]. Manual searching of the reference list of the relevant articles yielded 18 additional articles. After applying the inclusion criteria, eight of these articles were considered fit to include. Consequently, the combination of electronic and manual searching resulted in 36 articles which were included in the final review. To be specific, this review pooled the results of 15 human studies, consisting of 13 short-term randomized, controlled trials (RCT) and 2 cross-sectional studies, which had the assessed lipid-lowering effects of buckwheat in free-living subjects, and 21 animal studies. Nine human studies were conducted in China, two in India and one each in Sweden, Canada, Italy and Serbia. Ten animal studies were carried out in Japan, seven in China and one each in Spain, Poland, Egypt and South Korea.

### 3.2. Characteristics of Studies

Extracted data from the human and animal studies are in Table 1 and Table 2, respectively. All except two human cross-sectional studies in the review were RCT studies, with follow-up durations ranging from 7 days to 24 weeks in human studies and 10 days to 8 weeks in animal studies. Overall, buckwheat intake in RCT human studies ranged from 40 g to 300 g of buckwheat ingredients (median levels of individual series), with four studies not stating the amounts consumed. Participants were either healthy or had one or more CVD risk markers, including overweight, hypertension, hyperglycemia and hyperlipidemia. The methods of the included studies were similar, with a baseline period which was followed by subjects or animals being offered buckwheat or buckwheat-based products (e.g., buckwheat bread, buckwheat flour) for consumption, or placebo diets. Blood samples were obtained at baseline and after the intervention period for comparison of CVD biomarkers. It should be noted that outcomes in 12 out of 13 human intervention studies were only compared against baseline, and one study by Yu [23] was compared against a control group; removal of this study did not affect the outcomes of meta-analyses so it was retained in the dataset analyzed. Liver or feces were only available from animal studies. With respect to the two human cross-sectional studies, since the populations started to consume fairly high amounts of buckwheat seeds as a staple food from an early age, the outcomes obtained were adjudged as representing the long-term impact of buckwheat grain on CVD risk markers.

### 3.3. Human Studies

#### 3.3.1. Effects on Body Weight and BMI

Body weight or BMI changed significantly in response to buckwheat consumption in two out of seven human studies but in contrasting ways (Table 3). Body weight decreased by 3.44 kg among 44 overweight participants in one of the studies by Liu and Fu, while BMI was higher (estimated 3%) in consumers of buckwheat than in non-consumers in the study of Zhang and colleagues [18,28]. The other studies observed no significant impact of buckwheat consumption on body weight or BMI.

#### 3.3.2. Effects on Blood Pressure

The association between buckwheat intake and blood pressure yielded inconsistent results. Of six human studies which evaluated blood pressure, He and colleagues found that in those who consumed ≥40 g buckwheat/day, blood pressure was lower compared with those who consumed none or <40 g/day [36]. A significant reduction was also observed in hypertensive participants in the study conducted by Liu and Fu [28]. In a further study, systolic blood pressure was significantly decreased relative to the baseline, whereas diastolic blood pressure was only slightly, and not significantly lower in type 2 diabetic subjects [30]. For the remaining three human studies, there were no significant changes in blood pressure in response to intake of buckwheat-based foods.

#### 3.3.3. Effects on Blood Glucose and Insulin

**Figure 2:**
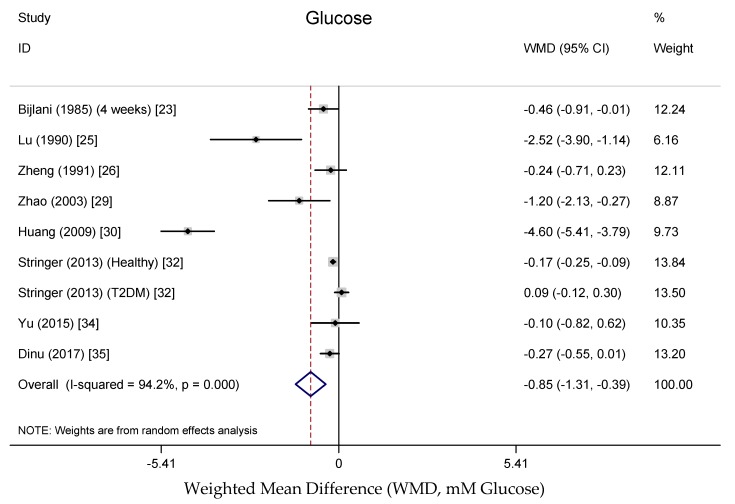
Meta-analysis of the effects of buckwheat products intake on blood glucose concentration compared with baseline or control groups for human studies. Sizes of data markers indicate the weight of each study in the analysis. WMD, weighted mean difference (the results were gained from a random-effects model). Negative values favor a reduction in blood glucose with buckwheat consumption; the dashed line shows the overall WMD value and the size of the rhombus the cumulative effect size and CI.

Data on fasting blood glucose concentrations were reported in nine randomized, controlled trials representing 548 participants based on the results of the meta-analysis. Figure 2 shows the pooled results from the random-effects model combing the weighted mean difference (WMD) for the impact of buckwheat intake on fasting glucose concentration in the total study population. The results show that the fasting blood glucose concentration was significantly decreased with buckwheat treatment in comparison with baseline or control groups (WMD, −0.85 mmol/L; 95% CI: −1.31, −0.39; *P* < 0.001), with significant heterogeneity in the data (*I*^2^ = 94.2%). This finding is consistent with the result of Zhang and colleagues, who showed that fasting blood glucose concentration of people in a buckwheat-eating region of Mongolia was significantly lower (16.92%) than that of people in a non-buckwheat-eating region of the country [18]. There was no consistent effect of buckwheat on insulin concentrations reported, with a small non-significant reduction and a small non-significant increase in insulin concentrations reported in two studies [27,35].

#### 3.3.4. Effects on Lipid Profile

**Figure 3:**
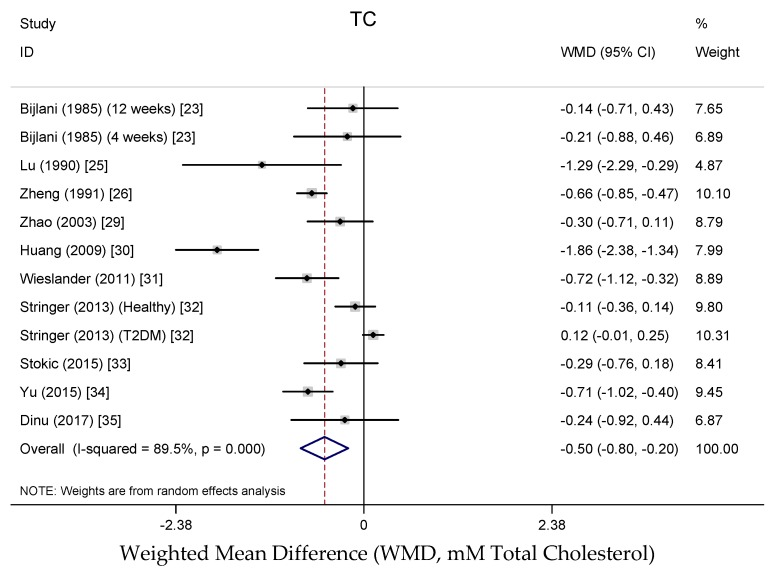
Meta-analysis of the effects of buckwheat products intake on blood total cholesterol concentration compared with baseline or control groups for human studies. Sizes of data markers indicate the weight of each study in the analysis. WMD, weighted mean difference (the results were gained from a random-effects model). Negative values favor a reduction in blood total cholesterol with buckwheat consumption; the dashed line shows the overall WMD value and the size of the rhombus the cumulative effect size and CI.

**Figure 4:**
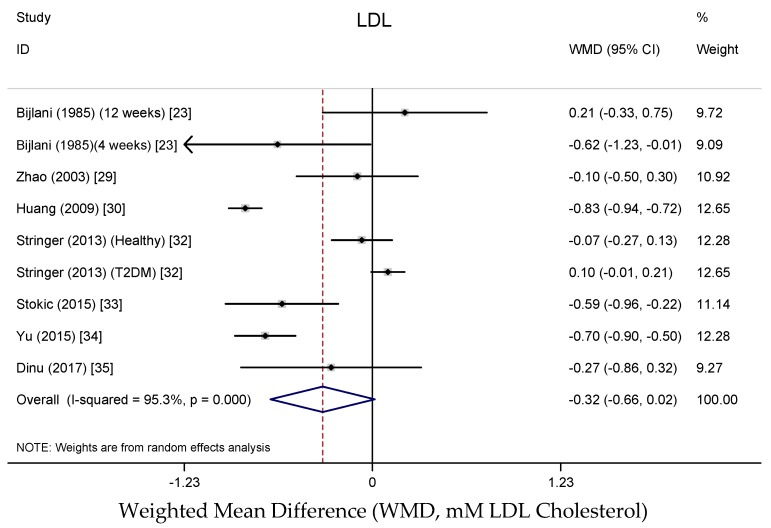
Meta-analysis of the effects of buckwheat products intake on blood LDL cholesterol concentration compared with baseline or control groups for human studies. Sizes of data markers indicate the weight of each study in the analysis. WMD, weighted mean difference (the results were gained from a random-effects model). Negative values favor a reduction in blood LDL cholesterol with buckwheat consumption; the dashed line shows the overall WMD value and the size of the rhombus the cumulative effect size and CI.

**Figure 5:**
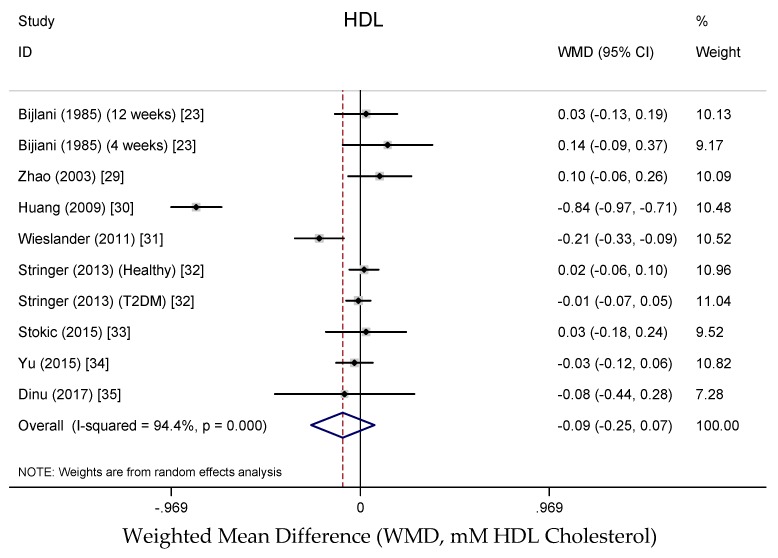
Meta-analysis of the effects of buckwheat products intake on blood HDL cholesterol concentration compared with baseline or control groups for human studies. Sizes of data markers indicate the weight of each study in the analysis. WMD, weighted mean difference (the results were gained from a random-effects model). Negative values favor a reduction in blood HDL Cholesterol with buckwheat consumption; the dashed line shows the overall WMD value and the size of the rhombus the cumulative effect size and CI.

**Figure 6:**
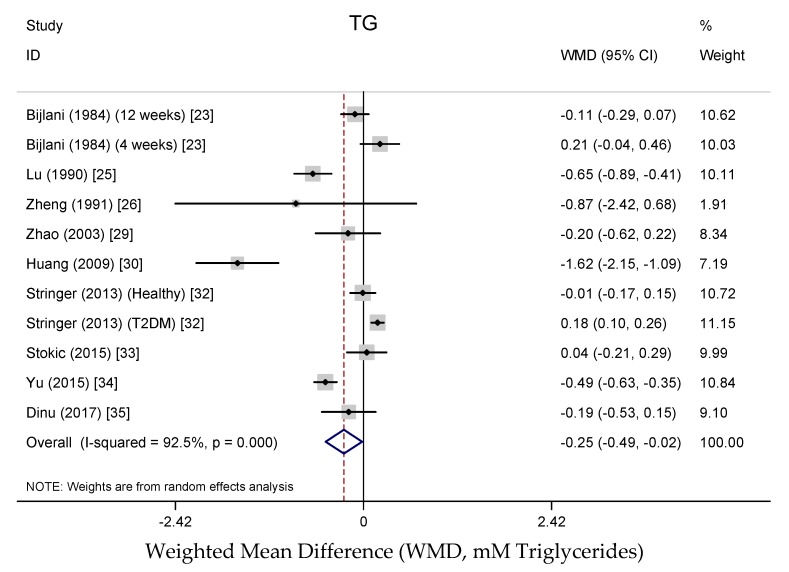
Meta-analysis of the effects of buckwheat products intake on blood triglycerides concentration compared with baseline or control groups for human studies. Sizes of data markers indicate the weight of each study in the analysis. WMD, weighted mean difference (the results were gained from a random-effects model). Negative values favor a reduction in blood triglycerides with buckwheat consumption; the dashed line shows the overall WMD value and the size of the rhombus the cumulative effect size and CI.

Results from the random-effects meta-analysis of the association between buckwheat intake and lipid parameters are shown in Figure 3, Figure 4, Figure 5 and Figure 6. Compared with baseline or control arms, buckwheat consumption was associated with statistically significantly lower concentrations of total cholesterol (WMD, −0.50 mmol/L; 95% CI: −0.80, −0.20; 12 trials, 708 participates, *I*^2^ = 89.5%, *P* = 0.001) and triglycerides (WMD, −0.25 mmol/L; 95% CI: −0.49, −0.02; 11 trials, 592 participates, *I*^2^ = 92.5%, *P* = 0.034). However, there were no significant effects on LDL-cholesterol (WMD, −0.33 mmol/L; 95% CI: −0.66, −0.02; 9 trials, 520 participates, *I*^2^ = 95.3%, *P* = 0.061) after buckwheat intake, nor on HDL-cholesterol (WMD, −0.09 mmol/L; 95% CI: −0.25, −0.07; 10 trials, 642 participates, *I*^2^ = 94.4%, *P* = 0.282).

In the cross-sectional study of 857 Yi men conducted by He and colleagues, after multiple-regression analysis, buckwheat intake (100 g/day) was associated with significantly lower concentrations of serum total cholesterol (−0.07 mmol/L, *P* < 0.01), LDL-cholesterol (−0.06 mmol/L, *P* < 0.05) and a higher ratio of HDL to total cholesterol (0.01, *P* < 0.05), with no effect on HDL-cholesterol and triglycerides [36]. These findings were in general accordance with the results from the trial by Zhang and colleagues with 961 participants, which also identified a significant decrease in HDL-cholesterol by 0.10 mmol/L (*P* < 0.01) [18].

#### 3.3.5. Sensitivity Analyses and Subgroups Analyses

In sensitivity analyses, after systematically removing individual studies, the beneficial pooled effects of buckwheat consumption on total cholesterol concentration were retained. However, the effect on triglycerides was no longer significant after removal of the study that had the largest effect on the overall result [31]. In contrast, the effect on LDL-cholesterol became statistically significant after the study that had the largest negative effects on the overall result was excluded [24]. No effects on glucose and HDL-cholesterol were observed when individual studies were removed (data not shown). Subgroup analyses were planned a priori to investigate whether study duration, buckwheat dose, types of buckwheat and study design altered the effects of buckwheat on glucose and lipid profiles, but the ability to do this was effectively hindered by the small numbers of studies for each trial, since meta-regression requires ≥10 studies per factor examined [58].

#### 3.3.6. Publication Bias

**Figure 7:**
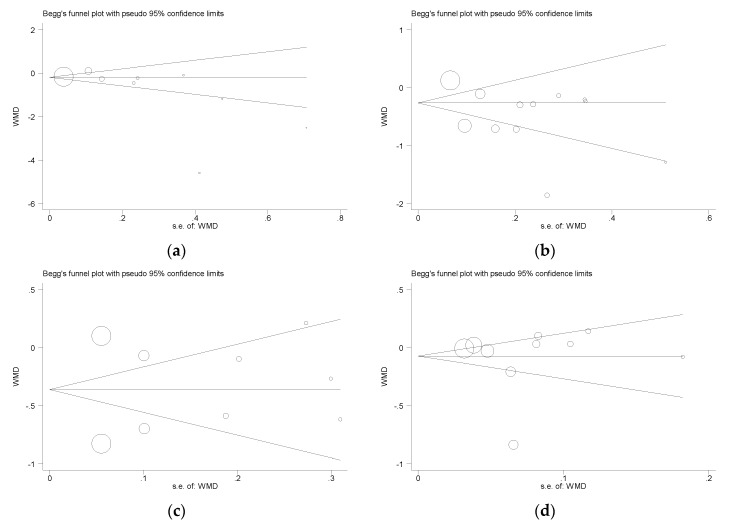

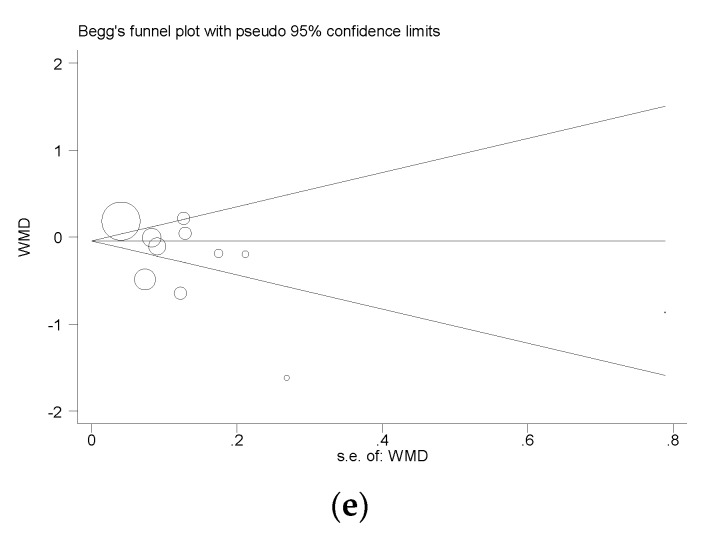
Publication bias funnel plots. Tests for publication bias of effects of buckwheat intake on (**a**) glucose, (**b**) total cholesterol (**c**) LDL cholesterol, (**d**) HDL cholesterol and (**e**) triglycerides. The funnel lines represent pseudo-95% confidence intervals; the size of the circles represent the weight of each study in the analysis. *p*-values (>0.05 for each plot) were derived from quantitative assessment of publication bias by Begg’s test and Egger’s test.

Funnel plots of the meta-analysis of the effect of buckwheat intake on glucose and lipid concentrations are shown in Figure 7. For glucose, Begg’s test *P* = 0.058, Egger’s test *P* = 0.130; for TC, Begg’s test *P* = 1.000, Egger’s test *P* = 0.089; for LDL, Begg’s test *P* = 1.000, Egger’s test *P* = 0.891; for HDL, Begg’s test *P* = 0.474, Egger’s test *P* = 0.720; TG, Begg’s test *P* = 0.350, Egger’s test *P* = 0.080) (Figure 7). Begg’s test and Egger’s test were not significant (*P* > 0.05), indicating that there was no evidence of publication bias.

### 3.4. Animal Studies

Because of the variability between species and study designs, data from animal studies were not pooled for meta-analysis but summarized below.

#### 3.4.1. Effects on Weight Gain and Food Intake

This review contains nineteen animal studies which reported the impact of buckwheat intake on body weight of which only four reported a significant decrease following buckwheat consumption, whereas one found a significant increase in body weight by 21.7% compared with the control [40]. With respect to the amounts of food consumed by the animals, food intake did not change significantly compared with that of the control group in twelve out of thirteen studies, while a marked increase in food intake was observed in the study by Tomotake and colleagues [47].

#### 3.4.2. Effects on Blood Glucose and Insulin

Three out of seven studies reported here showed a significant reduction in glucose concentration by between 15.2% and 18.4% (from 0.97 to 1.81 mmol/L), with the remaining studies showing that glucose concentration was unaffected by buckwheat treatment. With respect to blood insulin, insulin immunoreactivity was enhanced in one study, while a significant reduction in insulin concentration was observed in another study, and the two remaining studies found no significant changes.

#### 3.4.3. Effects on Lipid Profile

Of the twenty-one animal studies reported here, all investigated the impact of buckwheat intake on total cholesterol and seven reported results for LDL-cholesterol. Nineteen (90.5%) of the studies observed a significant reduction in total cholesterol and five (71.4%) of the studies observed a significant reduction in LDL cholesterol; the remainder identified no significant response. The significant decrease ranged from 11.7% to 54.1% (from 0.32 to 1.86 mmol/L) for total cholesterol and from 16.2% to 57.8% (from 0.22 to 1.24 mmol/L) for LDL-cholesterol. HDL cholesterol level increased from 19.6% to 54.6% (from 0.20 to 0.36 mmol/L) in four out of fourteen studies that reported this biomarker, while the level decreased (by between 11.5% and 28.4%) in another four studies. Of twenty animal studies analyzing the effect on triglycerides, all studies reported that intake of buckwheat consumption resulted in a fall in the serum concentration of triglycerides, which fell significantly (*P* < 0.05) from 2.3% to 73.9% (from 0.04 to 2.09 mmol/L) in fourteen studies.

#### 3.4.4. Other Outcomes

The liver weight of animals fed buckwheat food decreased significantly from 8.5 to 19.2% relative to the comparison group in eight out of eleven studies, while only one showed a significant increase by 5.4%. Eight of eleven studies found a reduction in liver total cholesterol content (*P* < 0.05), but no significant changes were detected in the other three studies. There was a significant increase in fecal weight and in fecal neutral steroids content by 57.6–171.0% and by 68.8–142.4% in five out of seven studies and all six studies, respectively.

## 4. Discussion

### 4.1. Effects on Body Weight

Being overweight brings about an elevated risk of health problems such as insulin resistance, type 2 diabetes mellitus, hypertension, hyperlipidemia and cardiovascular disease [59,60,61,62]. In order to evaluate the impact of buckwheat intake on body weight, the overall energy and macronutrient content in diets offered/consumed should be considered, but this was beyond the scope of this study. However, as mentioned above, there were few human and animal studies showing a significant reduction in body weight gain compared with baseline or control in response to consuming buckwheat-based food(s); restricted energy intake or intention to lose weight was not an intention of the studies reported.

Even though a significant reduction was observed in the study by Liu and colleagues, it must be noted that the participants involved in the study were overweight, and so body weight loss would not have been unexpected in an intervention study simply by engaging in the dietary intervention study itself [28]. Thus, on the basis of the published literature, it seems that the beneficial effects of buckwheat intake were not associated with weight loss, and this lack of association was consistent in both humans and animals with a variety of dietary levels of buckwheat or various forms of buckwheat products provided.

### 4.2. Effects on Blood Pressure

It is well known that hypertension is considered to be an important CVD risk factor, since half of ischemic heart disease and 60% of stroke cases are attributable to increased blood pressure [63,64]. One previous study revealed that 12 weeks intervention with whole grain (oats or oats with wheat) significantly lowered systolic blood pressure compared with a refined cereals group [65]. The effects of whole grain cereals on blood pressure, however, are inconsistent in comparison with observational data as reported by Seal and Brownlee and the paper from Tighe and colleagues is the only one to report a reduced blood pressure in a whole grain intervention that was not based on weight loss [65,66]. A significant reduction in blood pressure was only observed in one of the human studies reported here conducted by He and colleagues; these authors pointed out that water-soluble fibre, but not total dietary fibre, was independently associated with blood pressure and so an effect of buckwheat which has higher levels of soluble fibre than insoluble fibre is a possibility [36]. However, given the small number of studies carried out to date, this review is not adequately powered to conclude whether or not there are beneficial effects of buckwheat intake on blood pressure.

### 4.3. Effects on Blood Glucose and Insulin

Hyperglycaemia and insulin resistance are closely correlated to the risk of developing CVD [67,68]. There is considerable evidence showing that whole grain intake is associated with decreased glucose concentrations and is inversely associated with insulin resistance, suggesting that it is possible to regulate glucose and insulin homeostasis by cereal foods and their constituents [69,70,71]. Buckwheat is regarded as a low glycemic index (GI) food, and it has been demonstrated that low-GI diets significantly improved lipid profiles in medium- and long-term treatments, particularly with respect to decreasing both total and LDL cholesterol concentrations [72,73,74]. The results of animal studies with respect to the impact of buckwheat intake on glucose concentration, however, are conflicting, suggesting that results from animal studies do not strongly support the beneficial effects and may not be comparable to humans. In contrast, the meta-analysis of nine clinical trials indicated that diets supplemented with buckwheat were associated with a significant 0.85 mmol/L decrease in blood glucose concentration (*P* < 0.001). Of the many possible mechanisms responsible for modulating blood glucose concentrations, buckwheat is well known for containing various bioactive phytochemicals (such as various polyphenols and d-chiro-inositol), which have been shown to positively affect either glucose or insulin metabolism in animal models [75,76,77,78]. In addition, one study showed that the presence of resistant starch in buckwheat and buckwheat products contributed to its low glycemic index [79]. As for blood insulin, both human and animal studies yielded inconsistent results for the association between buckwheat intake and fasting blood insulin concentrations, indicating that there is no support for a beneficial effect of buckwheat on blood insulin or insulin-mediated glucose responses.

### 4.4. Effects on Lipid Profile

Cholesterol, produced in the liver and absorbed though the diet, is essential for all animal life in normal metabolic process. However, observational epidemiologic studies report that risk of heart attack in subjects with hyperlipidemia is three times higher than those in the general population with normal lipid status, while a 1% reduction in serum total cholesterol is strongly correlated with a 3% decrease in CVD risk [80,81]. Thus, treatments which are aimed at reducing cholesterol concentrations are effective in decreasing death risk from stroke and coronary heart disease. Consistent with two cross-sectional studies, this meta-analysis of the RCT studies indicated that increased intake of buckwheat-based products from 7 days to 27 weeks significantly improved an individual’s lipid profile, on average, decreasing total cholesterol by 0.50 mmol/L and triglycerides by 0.25 mmol/L. Moreover, the beneficial effects seen in human studies were also supported by strong evidence from animal studies, decreasing total cholesterol from 0.32 to 1.86 mmol/L and triglycerides from 0.04 to 2.09 mmol/L. Even though the change in LDL-cholesterol concentration was not statistically different (*P* = 0.061), the data approached statistical significance, and the mean reduction was 0.33 mmol/L, and significant decreases were also observed in two cross-sectional studies. It has been well known that a 1 mmol/L reduction of LDL-cholesterol lowers the morbidity and mortality of CVD patients by 22%, so a reduction of this magnitude could have significant clinical effects [82]. No effects of HDL-cholesterol were detected in the meta-analysis of RCT studies for buckwheat intake, in combination with inconsistent results from animal studies. The results of the meta-analysis were seen in both healthy and ‘‘at risk’’ subjects, but it is not possible within this review to examine differences in response between healthy and ‘‘at risk’’ subjects because of lack of power and the limited number of studies available. Nevertheless, it should be noted that one meta-analysis which investigated the effect of oats and oat-based products on lipid biomarkers, demonstrated that greater reductions were observed in studies where subjects initially had higher total cholesterol concentrations (>5.9 mmol/L) [83]. Thus, there was an indication that observed effects were generally more marked in subjects with higher CVD risk.

### 4.5. Buckwheat Intake Levels

Any evaluation of health benefits associated with food products should include an attempt to define optimal amounts for human consumption. The study of Liu and Fu, described in Table 1, showed that 40 g/day Tartary buckwheat flour for 4 weeks significantly lowered total cholesterol, LDL cholesterol and triglycerides concentrations compared with baseline [28]. The dose needed to reach a significant effect was similar to that of a large population-based study by He and colleagues, who found that buckwheat intake (≥40 g/day) was inversely related to markedly lower lipid profiles in comparison with those who consumed less than 40 g buckwheat/day [36]. Stringer and colleagues found that a higher amount of buckwheat cracker (containing buckwheat 76 g/day) for a shorter time period (7 days) did not significantly affect lipid profiles when compared with baseline, and similar results were also observed in two studies with longer intervention periods (4 and 12 weeks) by Bijlani and colleagues in 1984 and 1985 [24,25,33]. Studies showing the specific amount of buckwheat used are scarce, and more well-designed dose–response studies are required to confirm the minimum amounts of buckwheat needed to have a beneficial effect.

### 4.6. Bioactive Compounds Responsible for Lipid-Lowering Activity

The lipid-lowering activity of buckwheat has been ascribed to its nutritional composition including soluble fibre, protein, rutin and quercetin. However, due to the complexity of this composition, it is difficult to explore potential mechanisms underlying the beneficial effect of buckwheat on CVD risk. Some have been proposed but not fully explained, and it is possible that a combination of these components have contributed to the effects, instead of a single factor. As remarked previously, buckwheat is a good source of dietary fibre (5–11%), particularly the soluble fraction, which may help lower total cholesterol concentrations in the body [11,84,85]. The cross-sectional study by He and colleagues demonstrated that both total dietary and water-soluble fibre from buckwheat were significantly and independently correlated with lower serum total cholesterol concentrations, even though the average cholesterol concentration was low in the study population [36]. This result was in agreement with the results showing a similar correlation between water-soluble fibre and serum total cholesterol [37]. The cholesterol-lowering effects of soluble fibre may be accounted for by several mechanisms. It has been proposed that soluble fibre binds strongly to bile acids in the small intestine and elevates fecal bile acids excretion. The loss of bile acids in the stool stimulates the liver to increase cholesterol uptake from the circulation to replenish the bile acid supply. It also lowers the availability of bile acids for optimal fat digestion and absorption [86,87,88,89]. In addition, soluble fibre delays gastric emptying, slowing access of nutrients to digestive enzymes and to absorptive surfaces of the small intestine [90]. In addition, there is also emerging evidence that soluble fibre and resistant starch are additionally fermented by some bacteria in the colon, producing short-chain fatty acids (SCFA) perhaps via the inhibition of hepatic cholesterol synthesis in the liver, which helps to lower cholesterol concentrations [91,92]. One other mechanism that contributes to the cholesterol-lowering effects may be due to the low glycemic index of buckwheat in humans with the presence of resistance starch in the cereal [79,93]. However, the hypocholesterolemic effect of buckwheat starch, which was extracted from buckwheat flour, was not detected in rats when compared with corn starch [47].

It has been generally recognized that plant proteins may reduce plasma cholesterol concentrations, and the underling mechanisms of the cholesterol-lowering properties of plant proteins have been extensively analyzed [94,95,96]. However, in most studies, the effect of plant dietary proteins has focused on soybean protein, leading to limited information on the influence of other plant proteins and buckwheat proteins specifically on cholesterol metabolism. Despite having a relatively low digestibility, buckwheat protein, which accounts for 10% to 12.5% of flour weight, is an excellent supplement to other common grains, as it contains a good balance of amino acids with high nutritional value [9,97,98,99]. Previous studies have demonstrated a potent hypocholesterolemic activity of isolated buckwheat protein products prepared from buckwheat flour in rats or hamsters fed cholesterol-enriched or cholesterol free diets, which appeared to be stronger than that of soy protein isolate [47,48,49,50,51,52,53]. One study by Kayashita and colleagues further suggested that suppressive effects on cholesterol were mediated by enhanced excretion of fecal neutral sterols and that lower digestibility of buckwheat protein products is at least in part responsible for the effect. The lower digestibility may result in lower gastrointestinal transit time, which in turn leads to a higher stool weight and greater fecal excretion of neutral sterols. It has been observed that fecal excretion of neutral sterols was inversely correlated with serum cholesterol (r = −0.83, *P* < 0.01) [48]. Taken together, these impacts on rats appear to be similar to the properties of dietary fibre in humans [100,101]. To demonstrate this, Kayashita and colleagues also performed another experiment showing that plasma cholesterol in rats fed intact buckwheat protein products for two weeks was significantly lower than that in rats fed trypsin-digested protein. Moreover, this hypothesis has confirmed that, in the human body, the digestibility of buckwheat seed proteins was relatively low, owing possibly to the existence of phytic acid, tannins and protease inhibitors [102]. However, this seemed to contrast with the results that Tartary buckwheat had a reduced cholesterol-lowering impact on rats compared with common buckwheat, even though the digestibility of Tartary buckwheat was lower than that of common buckwheat [48]. It is noteworthy that human digestion is hugely different from that of rodents, such as rat and hamster, indicating that these results need to be interpreted with caution and more studies are required to answer this question [103]. In addition, the strong suppression of cholesterol by buckwheat protein products could be ascribed to its effect on higher bile acid synthesis, and also a greater excretion of fecal bile acids observed in rats, with the possibility that buckwheat protein products could possess some bile acid-binding proteins [47,49]. It has been further demonstrated in vitro that digestion-resistant peptides were largely responsible for bile acid binding activity of buckwheat protein digests and bile acid elimination [104,105]. Consistent with this, Zhang and colleagues very recently further suggested that Tartary buckwheat protein was one of the active ingredients to decrease plasma total cholesterol concentration, mainly regulated by improving the excretion of bile acids by its effects on the gene expression of hepatic CYP7A1 in an uptrend, but also preventing absorption of dietary cholesterol by its effects on the gene expression of intestinal Niemann-Pick C1-like protein 1 (NPC1L1), acyl CoA: cholesterol acyltransferase 2 (ACAT2), and ATP binding cassette transporters 5 and 8 (ABCG5/8) in a downtrend [57]. Moreover, the composition of amino acids in dietary proteins might be another important factor influencing blood cholesterol concentration, especially the ratio of lysine to arginine, which is even lower in buckwheat protein than that of soy protein [51]. Thus, it has been speculated that the cholesterol-lowering effect of buckwheat protein products observed may be ascribed to lower lysine: arginine ratio [51]. However, this hypothesis did not support the results that plasma cholesterol was unaffected with the addition of arginine in the diets [50].

It is well known that Tartary buckwheat seeds are a major source of rutin and quercetin [106]. Minor amounts of quercetin identified in Tartary buckwheat seeds are the result of rutin degradation [107,108]. The possibility of buckwheat rutin being one of the active components responsible for the suppressive effect on cholesterol concentrations cannot be eliminated. Buckwheat is well known to contain high concentrations of rutin (estimated 1.14%), a unique high flavonoid content compared with other common plant foods. Rutin has been shown to prevent the increase of plasma total cholesterol and non-HDL cholesterol in rats or mice fed with a high cholesterol or high fat diet [56,109,110,111,112]. However, in contrast to the results with rats and mice, serum total cholesterol concentrations in day-care staff were found to be lower in response to consuming cookies prepared from common or Tartary buckwheat, but no significant differences were detected between two buckwheat groups, even though the rutin content in Tartary buckwheat seed was 30 to 150 times greater than that in common buckwheat [32,113]. It has also been suggested that the cholesterol-lowering effects seen in animal models may be partially attributable to the quercetin content in buckwheat. In animal models (rat, rabbit, and mice) fed a high-cholesterol or high-fat diet, dietary quercetin given has been demonstrated to lower serum total cholesterol concentration [114,115,116]. However, the results regarding the effects of quercetin on cholesterol concentrations are controversial; several studies have reported that quercetin intake had no significant beneficial effects on total, LDL or HDL cholesterol and triglycerides [117,118,119,120]. The underling mechanisms of the quercetin on lipid metabolisms may be accounted for by the inhibition of cholesterol synthesis in hepatocytes and also the enzyme myeloperoxidase which was shown to oxidize lipoproteins [121,122,123].

### 4.7. Sensitivity Analysis

In the sensitivity analyses, removing individual studies systematically retained the statistical significance of the effects of buckwheat on total cholesterol, supporting the stability of the observed effects, but the effect on triglycerides was no longer significant possibly due to reduced statistical power. This finding indicates that effects on triglycerides were not stable to sensitivity analysis, in which individual studies were removed, thus, such analyses should be interpreted with more caution.

### 4.8. Limitations

Several limitations of this review should be noted. Firstly, relatively few long-term randomized and well-controlled human studies have directly investigated the effects of buckwheat intervention on risk markers for CVD, including weight gain, blood pressure, fasting blood glucose, insulin and lipids, and studies to date have been of short duration with small sample sizes. In order to support the effects, further, more large-scale, long-term human intervention studies are required. Secondly, there was considerable variability in study design between studies included in the meta-analyses, with different study durations and dose of buckwheat consumed. These effects may be taken into account in regression meta-analysis; testing the number of studies available for such analyses was considered insufficient. Whilst the results from the studies were largely consistent, and removal of individual studies in sensitivity analyses showed that no study overly impacted on the results; this suggests that the results should be treated with caution. Thirdly, most animal studies performed to date have analyzed the effect of individual molecular components or various buckwheat extracts on cell lines and animal models. However, human beings consume entire buckwheat seeds (as flour in products) instead of individual extracts, leading to uncertainty regarding whether the efficacy can be extrapolated to human health without further evaluation. Finally, the bioactive compounds responsible for buckwheat’s cardiovascular health still remain uncertain, and the mechanisms underlying the effects were not fully elucidated.

## 5. Conclusions

In conclusion, even though the literature to date is limited and often inconsistent in terms of study results, this review suggests that increased intake of buckwheat may lower CVD risk markers, including glucose, total cholesterol and triglycerides. Therefore, buckwheat, being a gluten-free alternative to some common grains, such as wheat, barley and rye, deserves to be a part of our daily diet. However, it still remains unclear whether increased intake of buckwheat has significant impacts on some CVD risk markers such as body weight and LDL cholesterol. There is increasing evidence that reduction in some risk markers associated with CVD could be due to polyphenol (phytochemical), soluble fibre, protein, rutin, quercetin and other components in the buckwheat, but it has not been fully elucidated which bioactive compounds are responsible for the underlying effects. Further research, especially large, well-powered, long-term human intervention studies, are required to further understand and promote the role that buckwheat seeds can play in cardiovascular health.

## Figures and Tables

**Table 1:** Summary of all human studies reviewed.

Human Studies						
Source	Study Population	Foodstuff; Intake	Duration	Outcomes 95% CI		
				Significant	Insignificant	
Bijlani et al. (1985) [24]	healthy (*n* = 8♂)	100 g of whole BW flour	12 weeks	serum: VLDL	↓	body weight
						serum: TC
						serum: LDL
						serum: HDL
						serum: HDL/TC
						serum: LDL_TG_
						serum: VLDL_TG_
						serum: HDL_TG_
						serum: TG
	healthy (*n* = 9♂)	100 g of whole BW flour	4 weeks	serum: HDL/TC	↑	body weight
				serum: LDL_TG_	↑	fasting blood glucose
				serum: VLDL_TG_	↑	glucose
				serum: HDL_TG_	↓	serum: TC
						serum: LDL
						serum: VLDL
						serum: HDL
						serum: TG
Bijlani et al. (1984) [25]	healthy (*n* = 12♂)	100 g of sieved BW preparation	4 weeks	serum: HDL	↑	blood glucose
				serum: HDL/TC	↑	serum: TC
						serum: LDL
						serum: VLDL
Lu et al. (1990) [26]	patients with diabetes and hyperlipidemia (*n* = 23, 13 and 18)	BW flour	1 month	fasting blood sugar	↓	
		BW flour	1 month	serum: TC	↓	
		BW flour	1 month	serum: TG	↓	
Zheng et al. (1991) [27]	NIDDM patients (*n* = 10♂, 9♀)	Tartary BW flour; 50 g	3 months	serum: TG	↓	Blood glucose
						insulin
						serum: TC
Liu and Fu (1996) [28]	patients (*n* = 60)	Tartary BW flour; 40 g/day	4 weeks	body weight	↓	
				systolic BP	↓	
				diastolic BP	↓	
				serum: TC	↓	
				serum: LDL	↓	
				serum: HDL	↑	
				serum: TG	↓	
Lin et al. (1998) [29]	Type 2 diabetes (T2DM) (*n* = 32)	100 g of Tartary BW flour	5 weeks	fasting blood	↓	serum: TC
				glucose		
				serum: TG	↓	
Zhao and Guan (2003) [30]	T2DM (*n* = 30♂, 30♀)	BW flour	8 weeks	blood glucose	↓	body weight
				systolic BP	↓	diastolic BP
				serum: TC	↓	serum: LDL
				serum: HDL	↓	serum: TG
Huang et al. (2009) [31]	patients with diabtes (*n* = 18♂, 17♀)	Tartary BW mixture	2 months	blood glucose	↓	
				HbA_1_ c/%	↓	
				serum: TC	↓	
				serum: LDL	↓	
				serum: HDL	↓	
				serum: TG	↓	
Wieslander et al. (2011) [32]	healthy (*n* = 62♀)	group 1: four common BW cookies (daily). group 2: four Tartary BW cookies (daily). (after 2 weeks wash-out, the groups switch type of cookies) 100 g of sieved BW preparation	6 weeks	serum: TC	↓	sPLA_2_
				serum: HDL	↓	
			
			
			
			
			
Stringer et al. (2013) [33]	healthy (*n* = 23)	BW cracker; 76 g	7 days			plasma glucose
						plasma: TC
						plasma: LDL
						plasma: HDL
						plasma: TG
						liver enzyme AST
						liver enzyme ALT
	T2DM (*n* = 24)	BW cracker; 76 g	7 days			plasma glucose
						plasma: TC
						plasma: LDL
						plasma: HDL
						plasma: TG
						liver enzyme AST
						liver enzyme ALT
Stokić et al. (2015) [34]	patients (*n* = 7♂, 13♀)	BW-enriched wheat bread; 300 g/day	1 month	serum: TC	↓	BMI
				serum: LDL	↓	systolic BP
				liver enzyme AST	↑	diastolic BP
				liver enzyme ALT	↓	serum: HDL
						serum: TG
Yu (2015) [23]	patients with hyperlipidemia (*n* = 36♂, 24♀)	Tartary BW tea, 15 g	60 days	serum: TC	↓	systolic BP
				serum: LDL	↓	diastolic BP
				serum: TG	↓	blood glucose
						serum: HDL
Dinu et al. (2017) [35]	participants with high CVD risk (*n* = 10♂, 11♀)	group 1: BW products (daily); group 2: control products (daily) (after 8 weeks wash-out, the groups switch type of products)	24 weeks	fasting blood glucose	↓	body weight
				glucose		insulin
				serum: TC	↓	serum: HDL
				serum: LDL	↓	
				serum: TG	↓	
He et al. (1995) [36]	healthy (*n* = 857♂)	BW; group 1 (*n* = 319), 0 g/day group 2 (*n* = 207), <40 g/day group 3 (*n* = 161), 40–200 g/day group 4 (*n* = 163), >200 g/day	cross-sectional study	systolic BP	↓	BMI
				diastolic BP	↓	serum: HDL
				serum: TC	↑	serum: TG
				serum: LDL	↓	
				serum: HDL/TC	↓	
Zhang et al. (2007) [18]	healthy (*n* = 491♂, 470♀)	BW; not stated	cross-sectional study	BMI	↑	systolic BP
				blood glucose	↓	diastolic BP
				serum: TC	↓	serum: TG
				serum: LDL	↓	
				serum: HDL	↑	

BW, buckwheat; VLDL, very low-density lipoprotein; TC, total cholesterol; LDL, low-density lipoprotein; HDL, high-density lipoprotein; TG, triglycerides; BP, blood pressure; HbA1 c, glycated hemoglobin A1c; sPLA2, secretory phospholipase A2; AST, aspartate transaminase; ALT, alanine transaminase; BMI, body mass index.

**Table 2:** Summary of all animal studies reviewed.

Animal Studies						
Source	Model	Assay Product; Dose	Duration	Outcomes 95% CI		
				Significant		Insignificant
Son et al. (2008) [37]	♂Sprague–Dawley rats	BW powder; 50% in the diets (diet with 1% cholesterol)	4 weeks	plasma: TC	↓	food intake
				plasma: LDL	↓	body weight gain
				plasma: HDL	↑	food efficiency ratio
				plasma: TG	↓	transit time
				area of lumen	↑	wall thickness
Yang et al. (2014) [38]	♂Syrian Golden hamster	Tartary BW flour; 24% in diet (fed cholesterol diet)	6 weeks	serum: TC	↓	food intake
				serum: non-HDL	↓	body weight gain
				liver cholesterol	↓	serum: HDL
				feces: neutral sterols	↑	serum: TG
						feces: acidic sterols
Prestamo (1985) et al. [39]	♀Wistar Hannover rats	cooked BW	30 days	body weight	↓	blood glucose
				serum: TC	↓	serum: LDL
				serum: HDL	↓	serum: TG
				HDL phospholipids	↓	liver weight
						uric acids
Orzel et al. (2015) [40]	♂Wistar rats	buckwheat flour, meal and bran; 200 g/kg (normal diet)	4 weeks	body weight gain	↑	food intake
				serum: LDL	↓	glucose
				serum: TG	↓	hemoglobin
						serum: TC
						serum: HDL
Tomotake et al. (1985) [41]	♂Sprague–Dawley rats and ♂ddY mice	30.7% of BWP extract in the diet (rats fed a normal or high-cholesterol diet); 54.8% of PBF (mice fed a high-cholesterol diet)	10 or 27 days	serum: TC	↓	food intake
				serum: TG	↓	body weight gain
				serum: phospholipids	↓	
				liver weight	↓	
				liver cholesterol (PBF)	↓	
				feces: dry weight (PBF)	↑	
				feces: neutral steroids	↑	
				feces: bile acids (PBF)	↑	
Magdy et al. (2014) [42]	♂albino rats	BW hull extracts; 1000 mg/kg b. wt/day in diet (hypercholesterolemia-induced diet)	8 weeks	blood glucose	↓	plasma: HDL
				plasma: TC	↓	
				plasma: LDL	↓	
				plasma: TG	↓	
				plasma: AST	↓	
				plasma: ALT	↓	
Wang et al. (2009) [43]	♂pathogen-free Wistar rat	Tartary BW bran extract; 0.2–1 g/kg body weight (high-fat diet)	6 weeks	serum: TC	↓	body weight gain
				serum: HDL (low dose)	↑	serum: LDL
				serum: TG	↓	
				hepatic: TC	↓	
				hepatic: TG	↓	
Hosaka et al. (2014) [44]	KK-Ay mice	common BW bran powder; 0.05 mg/g body weight	6 weeks	body weight gain	↓	food intake
				serum: TG	↓	fasting blood glucose
				liver weight	↓	insulin resistance
						serum: TC
Yao et al. (2008) [45]	♂C57BL/6 control mice and diabetic KK-Ay mice	d-Chiro-Inositol (DCI) enriched Tartary BW bran extract (TBBE); 45–182 mg of TBBE/kg in diet	5 weeks	fasting blood glucose level	↓	body weight gain
				plasma: TG (high dose)	↓	plasma: TC
				Insulin immunoreactivity	↑	
				immunoreactivity		
Hu et al. (2015) [46]	♂Kunming mice	d-Chiro-Inositol (DCI) enriched Tartary BW extract (DTBE); 40, 80 and 160 mg per kg body weight/day (high-fructose water)	8 weeks	body weight gain	↓	all parameters in the group of 40 mg per kg body weight/day showed on significant effect except serum AST activity
				serum: glucose	↓	
				serum: insulin level	↓	
				serum: TC	↓	
				serum: LDL	↓	
				serum: HDL	↑	
				serum: TG	↓	
				liver weight	↓	
				serum AST activity	↓	
				serum ALT activity	↓	
Tomotake et al. (2000) [47]	♂Golden Syrian hamster	BWP extract; 381 g/kg (high-cholesterol diet)	2 weeks	food intake	↑	body weight gain
				plasma: TC	↓	hepatic TG
				plasma: HDL	↓	hepatic phospholipids
				plasma: HDL/TC	↑	
				plasma: TG	↓	
				plasma: phospholipids	↓	
				liver weight	↑	
				hepatic cholesterol	↓	
				fecal dry weight	↑	
				feces: neutral steroids	↑	
				feces: acidic steroids	↑	
Tomotake et al. (2007) [48]	♂Sprague–Dawley rats	Tartary BW flour protein and common BWP extract; 30.7% of common BWP and 43.7% of Tartary BWP in the diet (high-cholesterol diet)	27 days	serum: TC	↓	body weight gain
				liver weight	↓	food intake
				hepatic cholesterol	↓	
				fecal dry weight	↑	
				fecal excretion: nitrogen	↑	
				feces: neutral steroids	↑	
				feces: bile acids	↑	
				protein digestibility	↓	
Tomotake et al. (2001) [49]	♂Sprague–Dawley rats	BWP extract; 307 g/kg (normal diet)	8 weeks	plasma: TC	↓	body weight gain
				plasma: HDL	↓	food intake
				feces: neutral steroids	↑	plasma: TG
				feces: acidic steroids	↑	plasma: phospholipid
						feces dry weight
Kayashita et al. (1997) [50]	♂Sprague–Dawley rats	BWP extract; 381 g/kg (high-Cholesterol diet)	3 weeks	plasma: TC	↓	body weight gain
				plasma: HDL/TC	↑	food intake
				plasma: TG	↓	plasma: HDL
				plasma: phospholipids	↑	hepatic: TG
				plasma: bile acids	↓	feces: acidic steroids
				liver weight	↓	
				hepatic cholesterol	↓	
				hepatic: phospholipids	↓	
				feces dry weight	↑	
				feces: neutral steroids	↑	
				protein digestibility	↓	
Kayashita et al. [51]	♂Sprague–Dawley rats	BWP extract; 38.1%	3 weeks	plasma: TC	↓	body weight gain
				plasma: HDL/TC	↑	food intake
				plasma: TG	↓	plasma: HDL
				plasma: free fatty acid	↓	hepatic cholesterol
				plasma: phospholipids	↓	hepatic TG
				liver weight	↓	hepatic phospholipids
				fat pad weights	↓	
Kayashita et al. [52]	♂Sprague–Dawley rats	BWP extract; 381 g/kg	3 weeks	plasma: TC	↓	body weight gain
				hepatic TG	↓	food intake
				fecal dry weight	↑	insulin
				fat pad weights	↓	plasma: TG
						plasma: free fatty acid
						plasma: phospholipids
						liver weight
						hepatic TC
						hepatic phospholipids
Kayashita et al. [53]	♂Sprague–Dawley rats	BWP extract; 323.1 g/kg (high-Cholesterol diet)	3 weeks	plasma: TC	↓	body weight gain
				hepatic: weight	↓	food intake
				hepatic TC	↓	serum: TG
				hepatic TG	↑	serum: free fatty acids
						serum: glucose
Hu et al. [54]	♂Kunming mice	Tartary buckwheat flavonoid fraction; 200, 400 and 800 mg per kg bw in diet (high trimethylamine-*N*-oxide diet)	8 weeks	body weight gain	↓	food intake
				serum: TC	↓	water intake
				serum: LDL	↓	
				serum: HDL	↑	
				serum: TG	↓	
				liver weight	↓	
				hepatosomatic index	↓	
Han et al. [55]	Wister mice	total flavones of buckwheat seeds; 2 g/kg/day (high-fat diet)	10 days	serum: TC	↓	fasting blood glucose
				serum: TG	↓	
Qu et al. [56]	♂Sprague–Dawley rats	high rutin in BW noodles; 980 mg/kg in diet (high-fat, high-sucrose diet)	4 weeks	serum: TC	↓	body weight gain
				liver lipid	↑	feed efficiency
						serum: HDL
						serum: TG
						serum: free fatty acids
						liver TC
						dry weight of feces
						fecal total lipid
Zhang et al. [57]	♂Golden Syrian Hypercholesterolemia hamster	Tartary BWP extract; 353 g/kg in diet	6 weeks	plasma: TC	↓	body weight
				plasma: non-HDL	↓	fatty streak (%)
				plasma: HDL	↓	
				plasma: TG	↓	
				liver cholesterol	↓	
				total neutral sterols	↑	
				acidic sterols	↑	

BW, buckwheat; TC, total cholesterol; LDL, low-density lipoprotein; HDL, high-density lipoprotein; TG, triglycerides; BWP, buckwheat protein; PBF, protein buckwheat flour; AST, aspartate transaminase; ALT, alanine transaminase.

**Table 3:** The number of animal and human intervention studies showing significant increase, no effect and significant reduction on markers of CVD risk.

	Number of Studies		
	Significantly higher in Buckwheat Treatment	No Effect	Significantly Lower in Buckwheat Treatment
Human Studies			
Body weight gain or BMI	1	5	1
Blood pressure	—	3	3
Blood glucose	—	5	6
Blood insulin	—	2	—
Total-Cholesterol	—	5	10
LDL-Cholesterol	—	4	7
HDL-Cholesterol	3	6	3
Triglycerides	—	6	7
Animal Studies			
Body weight gain	1	14	4
Food intake	1	12	—
Blood glucose	—	4	3
Blood insulin	1	2	1
Total-Cholesterol	—	2	19
LDL-Cholesterol	—	2	5
HDL-Cholesterol	4	6	4
Triglycerides	—	6	14
Liver weight	1	2	8
Liver Total-Cholesterol	—	3	8
Fecal weight	5	2	—
Fecal neutral steroids	6	—	—
